# A group-mediated, home-based physical activity intervention for patients with peripheral artery disease: effects on social and psychological function

**DOI:** 10.1186/1479-5876-12-29

**Published:** 2014-01-28

**Authors:** W Jack Rejeski, Bonnie Spring, Kathryn Domanchuk, Huimin Tao, Lu Tian, Lihui Zhao, Mary M McDermott

**Affiliations:** 1 Departments of Health and Exercise Science and Geriatric Medicine, Wake Forest University, Box 7867, Winston-Salem, NC 27109, USA; 2 Feinberg School of Medicine, Northwestern University, Chicago, IL, USA; 3 Department of Health Research and Policy, Stanford University, Palo, Alto, CA, USA

**Keywords:** Peripheral artery disease, Group-mediated intervention, Physical activity, Social function, Psychological function

## Abstract

**Background:**

PAD is a disabling, chronic condition of the lower extremities that affects approximately 8 million people in the United States. The purpose of this study was to determine whether an innovative home-based walking exercise program for patients with peripheral artery disease (PAD) improves self-efficacy for walking, desire for physical competence, satisfaction for physical functioning, social functioning, and acceptance of PAD related pain and discomfort.

**Methods:**

The design was a 6-month randomized controlled clinical trial of 194 patients with PAD. Participants were randomized to 1 of 2 parallel groups: a home-based group-mediated cognitive behavioral walking intervention or an attention control condition.

**Results:**

Of the 194 participants randomized, 178 completed the baseline and 6-month follow-up visit. The mean age was 70.66 (±9.44) and was equally represented by men and women. Close to half of the cohort was African American. Following 6-months of treatment, the intervention group experienced greater improvement on self-efficacy (*p* = .0008), satisfaction with functioning (*p* = .0003), pain acceptance (*p* = .0002), and social functioning (*p* = .0008) than the control group; the effects were consistent across a number of potential moderating variables. Change in these outcomes was essentially independent of change in 6-minute walk performance.

**Trial registration:**

[ClinicalTrials.gov Identifier: NCT00693940]

## Introduction

Peripheral artery disease (PAD) is a disabling, chronic condition of the lower extremities that affects approximately 8 million people in the United States [1]. Leg pain caused by PAD compromises mobility and leads to faster rates of decline in mobility than in persons without the disease [2-4]. The Group Oriented Arterial Leg Study (GOALS) [5], a randomized controlled trial promoting home-based physical activity, recently demonstrated that 6-months of a group-mediated cognitive behavioral (GMCB) intervention improved 6-minute walk performance in patients with PAD. The current investigation focuses on changes in important outcomes that were targeted by the intervention including self-efficacy related to walking different distances at a brisk pace along with the desire to be able to complete these walking challenges, satisfaction with physical function, acceptance of PAD related pain/discomfort, and social functioning.

The conceptual and clinical significance of these outcomes in PAD is underscored by both existing theory [6,7] and applied research on the self-management of chronic disease in aging [8]. Older adults’ self-efficacy for physical functioning is a central dimension of well-being [9] and decline in physical function is inversely related to both the desire to engage in mobility related activities and to satisfaction with physical functioning [10]. As people self-manage the challenges related to living with PAD, social relations are important because they can provide guidance and emotional support, as well as enhance feelings of self-worth [7]. Finally, pain acceptance—the ability to engage in important life activities despite the pain and discomfort associated with the disease—is also important to managing PAD [11,12].

To summarize, the objective of this study was to evaluate the effect of the GOALS physical activity GMCB intervention on key psychological and social constructs. We hypothesized that individuals assigned to the physical activity intervention would improve on all outcomes compared to the control group after 6 months of treatment. This includes self-efficacy for walking, desire for physical competence, satisfaction for physical functioning, social functioning, and acceptance of PAD related pain/discomfort.

## Methods

The institutional review board of Northwestern University approved the protocol. Participants gave written informed consent. Methods for the GOALS trial have been reported [13]. The study was a parallel design randomized controlled clinical trial involving two groups: a home-based physical activity intervention or a health education attention control group. Data collection and study interventions were performed at Northwestern University Feinberg School of Medicine between 7/22/08 and 12/14/12.

### Participant identification

Participants were recruited through newspaper or radio advertisements or from mailed postcards to men and women age 65 and older living in the Chicago area. Additional details on recruitment have been previously reported [13].

### Inclusion and exclusion criteria

The inclusion criterion was an ankle brachial index (ABI) ≤ 0.90 in either leg. Potential participants with a resting ABI ≥ 0.91 and ≤ 1.00 at baseline were eligible if their ABI dropped by ≥ 20% following a heel-rise test. Potential participants with a resting ABI greater than 0.90 were eligible if they provided data from a certified vascular laboratory demonstrating prior lower extremity ischemia or if they had documented evidence from medical records of lower extremity revascularization. Potential participants with characteristics preventing full participation in an exercise intervention were excluded. These characteristics included a below or above-knee amputation, wheel-chair confinement, inability to walk at least 50 feet during the six-minute walk without stopping, use of a walking aid other than a cane, inability to return to the medical center for weekly study sessions, failure to complete the study run-in phase, walking impairment for a reason other than lower extremity ischemia, foot ulcer or critical limb ischemia, and significant visual or hearing impairment. Potential participants with characteristics that may influence study outcomes independent of study participation were also excluded. These characteristics included major surgery or lower extremity revascularization during the previous 3 months or planned during the next 12 months, major medical illness including cancer treatment during the prior 12 months, current participation in another clinical trial or participation in another exercise trial within the past three months, completion of cardiac rehabilitation during the past three months, Parkinson’s disease, and requiring oxygen with activity or exercise were excluded. Potential participants for whom exercise may be unsafe were excluded. These exclusion criteria included those with > Class II New York Heart Association heart failure or angina, an increase in angina pectoris during the prior six months, or an abnormal baseline exercise stress test were excluded. Finally, potential participants with a mini-mental status examination score < 23 at baseline were excluded because of concern that they may not respond well to the intervention and/or provide consistently accurate responses to study questionnaires [13].

### Measures

Outcomes measures were assessed before randomization and at six-month follow-up. Outcome examiners were blinded to participant group assignment.

#### Six-minute walk test

Following a standardized protocol, participants walked up and down a 100-foot hallway for six minutes after instructions to cover as much distance as possible. The distance completed after six minutes was recorded. We have previously reported the intraclass correlation coefficient for test/re-test reliability of the six-minute walk to be 0.90 (P < .001) when assessed 1–2 weeks apart (see [13] for complete details) [14].

#### Self-efficacy and desire for physical competence

The measures of walking-related self-efficacy and desire for physical competence consisted of responses to the same 10 walking-related items ranging from walking at a brisk pace for 300 feet without stopping to rest to walking at a brisk pace for 3 miles [12]. The self-efficacy measure required participants to respond to each item using an 11-point Likert-type scale ranging from 0 (not at all confident) to 10 (extremely confident), whereas the desire scale assessed participants desire to be able to complete each task described in the items ranging from 1 (low desire) to 4 (very strong desire). Psychometric support for the two measures has been previously published [12]. In brief, both measures are transformed to scales ranging from 0 to 100; they have acceptable levels of test-retest reliability (0.77 for self-efficacy and 0.66 for desire) and alpha internal consistency reliabilities (0.95 for self-efficacy and 0.97 for desire). The scales are conceptually independent (r = 0.13) and are related in expected directions to 6-minute walk performance.

#### Satisfaction with physical function

A 6-item measure originally developed by Ray and colleagues [15] was used to assess satisfaction with physical function. Each item is rated on a 7-point scale that is scored from −3 to +3 with the following phrases: very dissatisfied (−3), somewhat dissatisfied (−2), a little dissatisfied (−1), neither (0), a little satisfied (+1), somewhat satisfied (+2), very satisfied (+3). The measure has demonstrated good convergent validity and other psychometric properties [16] and has been used in several randomized controlled trials involving physical activity which found it sensitive to change in older adult populations [17,18].

#### Pain acceptance

In order to evaluate how the intervention affected the ability to cope with disease-related pain, we used a measure of pain acceptance originally developed by McCracken and colleagues [11] and subsequently modified for PAD by our research group [12]. Seven of the original items from each of two subscales (a total of 14 items) were worded in a manner appropriate for PAD: tolerance for pain during activities that cause pain/discomfort, and the feeling that pain/discomfort from PAD is highly disruptive and needs to be controlled. Factor analysis of the modified PAD measure supports the two-factor structure with test re-test reliabilities for the total and subscale scores all ≥ 0.70 [12]. As pain acceptance increased, time to complete a 6-minute walk test decreased supporting the construct validity of the measure [12].

#### Social functioning

To assess social functioning associated with involvement in the group-based interventions, we used a modified version of Cutrona and Russell’s Social Provision Scale (SPS) [7]. This measure consists of 24-items. A total score can be computed along with 6 subscales scores: guidance (advice or information), reliable alliance (assurance that others can be counted on in times of stress), reassurance of worth (recognition of one’s competence), attachment (emotional closeness), social integration (a sense of belonging to a group of friends), and opportunity for nurturance (providing assistance to others). Participants’ level of agreement with each item is assessed using a 4 point rating scale: strongly disagree = 1, disagree = 2, agree = 3 and strongly agree = 4. Using the Social Provisions Scale, scores can be derived for each of the 6 provisions as well as for a global social provisions score. Research has supported the reliability and validity of the SPS [7]. Scores on the measure have been shown to predict adaptation to stress among a wide variety of populations, including post-partum women, spouses of cancer patients, the elderly, and individuals working in stressful job situations (see http://ccutrona.public.iastate.edu/socprov.htm).

#### Ankle brachial index (ABI)

A handheld Doppler probe (Pocket Dop II; Nicolet Biomedical Inc.) was used to obtain systolic blood pressure twice in the right and left brachial, dorsalis pedis, and posterior tibial arteries using established methods. The ABI was calculated by dividing the mean of the dorsalis pedis and posterior tibial pressure levels in each leg by the mean of the 4 brachial blood pressures. Systolic blood pressure levels of zero were excluded from ABI calculations. Mean blood pressure levels in the arm with the higher pressure were used when one brachial pressure was higher than the opposite brachial pressure in both measurement sets and the 2 brachial pressures differed by 10 mm Hg or more in a single measurement set [5].

### Randomization

After baseline testing, eligible participants were randomized by computer using a randomly permuted block method. Randomization was stratified by baseline 6-minute walk performance in order to ensure an equal distribution of walking performance between the two study groups.

### Study interventions

The home-based physical activity GMCB intervention was designed to help participants adhere to daily walking exercise goals. Participants met once weekly in a group with other PAD participants, led by a trained facilitator. Sessions were approximately 90 minutes, with 45 minutes devoted to facilitator-led discussions and 45 minutes devoted to walking exercise around an indoor track at the exercise facility. Session topics have been reported [13] and included an overview of PAD, information on the benefits of walking exercise for PAD, goal-setting, self-monitoring, and managing pain during exercise. Participants were asked to complete a walking goal form each week on which they listed walking goals for at least five days of the week. Participants were encouraged to engage in over-ground (rather than treadmill) walking, since over-ground walking more closely simulates walking in daily life. Participants were advised to walk until they experienced severe leg discomfort (i.e. a severity of 4 or 5 on a scale of 0–5) and then rest until the leg discomfort subsided sufficiently to resume walking. Participants without leg symptoms were asked to walk to an intensity of 12–14 on the Borg Rating of Perceived Exertion (RPE) scale [19]. Participants recorded their actual walking exercise each day, severity of their leg pain/discomfort, and their RPE during each walking exercise session. The facilitator reviewed walking forms each week and provided brief individualized feedback. Individuals were asked to increase their walking activity over time with the goal of achieving 50 minutes of walking per session at least five days per week.

The health education attention control group attended weekly 60 minute group sessions with other PAD participants. Physicians and other health-care professionals provided educational information on health-related topics to the study participants. Topics included management of hypertension, cancer screening, preventing falls, and vaccinations. Exercise and behavior change were not discussed.

### Statistical analyses

Chi-square tests and one-way analyses of variance were used to compare characteristics of participants across the two groups at baseline. Two sample, two-sided t-tests were used to compare changes in outcomes between baseline and six-month follow-up between the intervention and the control group. Because there were 5 distinct outcomes, *a priori* the p value considered statistically significant was p <0.01. Intention-to-treat analyses were performed with all analyses performed using SAS version 9.2.

## Results

Of the 194 participants that were randomized to treatment, 178 completed the baseline and 6-month follow-up visit. Table 1 provides descriptive characteristics of the study cohort and illustrates the similarities of the intervention and control group at the time of baseline assessments. The mean age of the sample was 70.66 (±9.44) and was equally represented by men and women. Close to half of the cohort was African American and there were multiple comorbidities with diabetes and knee osteoarthritis having the highest prevalence; that is, 33.71% and 28.25, respectively. Roughly one-fifth of the cohort were current smokers and the average BMI was close to the cutpoint of 30 kg/m^2^ for class I obesity, with a mean of 28.79 (±6.54) kg/m^2^.

**Table 1:** Baseline characteristics of the sample*

**Baseline measures**	**Overall N = 178**	**Control group N = 90**	**Intervention group N = 88**	**p value**
Age	70.66 (9.44)	71.64 (9.51)	69.65 (9.32)	0.1589
Male, %	49.44	48.89	50.00	0.8822
African-American, %	48.88	42.22	55.68	0.0725
Ankle brachial index	0.67 (0.17)	0.68 (0.18)	0.67 (0.16)	0.5309
Body mass index (kg/m^2^)	28.79 (6.54)	29.13 (6.67)	28.45 (6.42)	0.4928
Current smoker, %	21.91	18.89	25.00	0.3244
Angina, %	16.38	15.73	17.05	0.8132
MI, %	13.48	14.44	12.50	0.7041
CHF, %	11.24	12.22	10.23	0.6735
Stroke, %	13.48	16.67	10.23	0.2085
Pulmonary disease, %	12.92	13.33	12.50	0.8684
Cancer, %	16.38	16.85	15.91	0.8652
Diabetes mellitus, %	33.71	37.78	29.55	0.2454
Knee arthritis, %	28.25	22.47	34.09	0.086
Hip arthritis, %	15.73	18.89	12.50	0.2418
Spinal stenosis, %	9.55	7.78	11.36	0.4157
Disc disease, %	23.73	24.72	22.73	0.7555
Rheumatoid arthritis, %	11.3	7.87	14.77	0.1467
Six minute walk (m)	355.33 (94.54)	353.27 (91.92)	357.43 (97.64)	0.7701

Legend: *All table values are unadjusted and reflect the mean (SD) unless specified as%. MI = myocardial infarction; CHF = congestive heart failure.

### Analyses of study outcomes

Table 2 provides the means (SD) for the social cognitive variables at baseline and 6-month follow-up for both the physical activity intervention and control group along with LS Means for group differences. For the primary analyses, which examined group differences for 5 of the 13 outcomes shown in Table 2 (self-efficacy, desire for physical competence, satisfaction with physical function, the summary pain acceptance score, and the summary score for social provisions) all measures except desire for physical competence improved in the physical activity intervention group as compared to the control condition; all p values < .01. Secondary analyses of the pain measure revealed that the activity subscale of the pain acceptance measure accounted for much of the observed treatment difference. In contrast, for social functioning, 4 of 6 subscales were improved in the physical activity intervention group as compared to the control group: guidance, reassurance of worth, attachment and nurturance.

**Table 2:** Group comparisons for social cognitive outcome variables: 0 to 6 months

**Measure**	**Group**	**N**	**Baseline mean (SD)**	**6-Month mean (SD)**	**LS mean group differences (95% CI)**	**P**
Self-efficacy	C	89	16.98 (14.73)	17.27 (15.58)	Reference	
	I	88	16.52 (16.54)	22.81 (15.34)	5.99 (1.56,10.42)	0.0083
Desire for physical function	C	90	21.12 (11.87)	20.19 (12.11)	Reference	
	I	88	22.09 (12.66)	23.99 (11.26)	2.83 (−0.63,6.29)	0.1079
Satisfaction with function	C	90	−0.49 (1.60)	−0.24 (1.63)	Reference	
	I	88	−0.52 (1.64)	0.43 (1.43)	0.70 (0.33,1.08)	0.0003
Pain acceptance	C	78	2.91 (0.80)	3.07 (0.75)	Reference	
	I	81	2.94 (0.77)	3.56 (0.77)	0.46 (0.22, 0.69)	0.0001
Pain: activity engagement	C	78	3.83 (1.20)	3.99 (1.17)	Reference	
	I	81	3.62 (1.06)	4.39 (1.20)	0.62 (0.25, 0.99)	0.0013
Pain: Willingness	C	78	1.99 (1.01)	2.17 (1.04)	Reference	
	I	80	2.24 (0.93)	2.73 (1.04)	0.31 (−0.02, 0.64)	0.0640
SPS: Total score	C	89	52.69 (8.63)	54.06 (9.08)	Reference	
	I	86	52.55 (7.90)	59.12 (9.48)	5.20 (2.12,8.19)	0.0008
SPS: Guidance	C	89	9.06 (2.18)	9.31 (2.12)	Reference	
	I	86	9.15 (1.96)	10.45 (2.06)	1.04 (0.28,1.81)	0.0081
SPS: Reassurance of worth	C	89	9.13 (1.53)	9.65 (1.56)	Reference	
	I	86	9.17 (1.31)	10.60 (1.60)	0.91 (0.37,1.45)	0.0011
SPS: Social integration	C	89	9.52 (1.68)	9.65 (1.83)	Reference	
	I	86	9.28 (1.57)	10.05 (1.92)	0.63 (−0.02,1.29)	0.0577
SPS: Attach	C	89	8.06 (2.09)	8.39 (2.34)	Reference	
	I	86	7.79 (2.00)	9.42 (2.02)	1.29 (0.52,2.06)	0.0011
SPS: Nurturance	C	89	7.27 (1.93)	7.03 (2.31)	Reference	
	I	86	7.21 (1.93)	7.84 (2.20)	0.86 (0.18,1.55)	0.014
SPS: Reliable alliance	C	89	9.65 (1.92)	10.01 (2.08)	Reference	
	I	86	9.94 (1.63)	10.76 (1.85)	0.45 (−0.18,1.09	0.1597

Legend: SPS = social provision scale; C = control; I = intervention.

**Figure 1:**
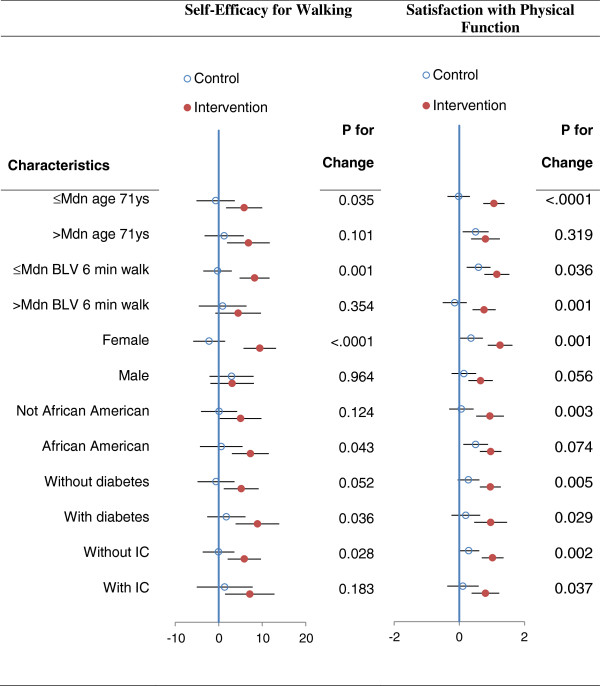
**Forest plots for treatment differences (±95 CI) on selected characteristics for self- efficacy and satisfaction with physical function.** Legend: Mdn = median; BLV = baseline visit; IC = intermittent claudication; the plotted points represent change from baseline for the control and intervention groups and the *p* values to the right are the probability values for the control versus treatment comparisons.

**Figure 2:**
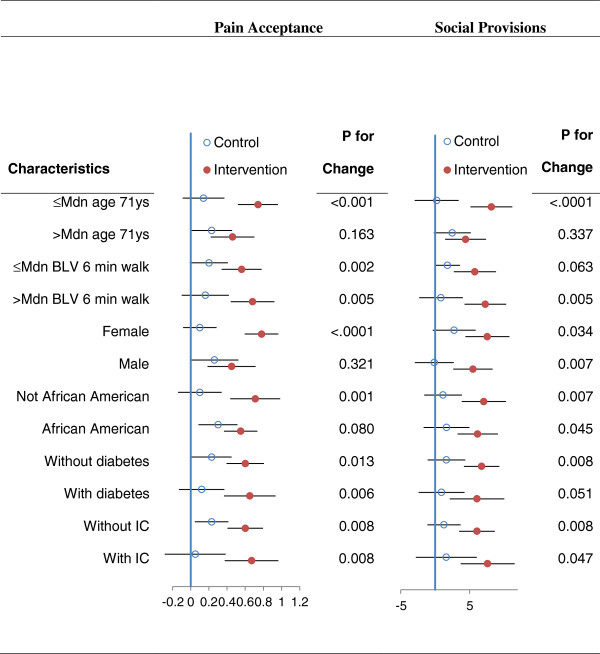
**Forest plots for treatment differences (±95 CI) on selected characteristics for pain acceptance and social provisions.** Legend: Mdn = median; BLV = baseline visit; IC = intermittent claudication; the plotted points represent change from baseline for the control and intervention groups and the *p* values to the right are the probability values for the control versus treatment comparisons.

Forest plots presented in Figures 1 and 2 illustrate that the treatment group differences on the four statistically significant outcomes were relatively comparable for most potential moderator variables. With the exception of the treatment by age group interaction for satisfaction with physical function, which just achieved statistical significance (p = 0.045) without any adjustment for multiple comparisons, none of the other 23 tests for interactions exceeded the *p* < .05 level of statistical significance. Also when plotted points in the plots seem to suggest moderation, in most instances these trends were due to positive change in the control group.

### Adherence, 6-min walk, physical activity, and serious adverse events

Both the control and intervention groups had similar levels of attendance to scheduled center-based contacts: 68.48% (±31.59%) for control and 71.71% (±28.78%) for intervention, *p* = 0.4577. As reported in the primary outcomes paper, [5] participants randomized to the intervention group significantly improved their 6-min walk performance at the 6-month follow-up visit by 53.5 meters [95% CI, 33.2 to 73.8] relative to the control group, *p* < .001; on average, they also had 114.7 more activity units per week than the control group at 6-month follow-up as reported by Accelerometry [95% CI, 12.82 to 216.50], *p* = .03. Within the exercise intervention there was only one SAE that we determined could have been related to the exercise intervention. A participant reported new dyspnea at the exercise session, was sent to the ER, and underwent a CABG.

We conducted Pearson Product Moment Correlations between change in each of the four statistically significant social cognitive outcomes with change in the primary outcome, change in the 6-minute walk. Only change in the total score for the social provision scale from baseline to 6-months had a relationship with change in the 6-minute walk and this effect was small and marginally significant, *r* = 0.21, *p* = 0.056.

## Discussion

Our findings expand upon the positive results reported in the GOALS primary outcomes paper [5] in which the GMCB intervention group achieved a statistically and clinically significant change in the 6-minute walk after 6 months of treatment as compared to a health education control condition. In the current study, the intervention group was found to experience statistically significant improvement in walking self-efficacy, satisfaction with physical function, pain acceptance, and social functioning. It is important to note that change in these outcomes was essentially independent of change in 6-minute walk performance, demonstrating that patients with PAD realize multiple benefits with this type of intervention. Also, as shown in the forest plots, the effects were consistent across a number of potential moderating variables. Change in these outcomes is consistent with the both the content/goals of the group-mediated intervention and is supported by other published work in this area [10,20].

These results are consistent with a growing body of literature on older adult populations with various chronic health conditions demonstrating that physical activity interventions enhance participants’ confidence in their capacity for performing various mobility-related activities [21,22]. The physical activity intervention also increased their satisfaction with physical function, [10] an important outcome expectation for older adults who participate in lifestyle interventions [20]. The positive effects observed for pain acceptance and social functioning in the intervention group are equally noteworthy. It is well known that diseases accompanied by chronic pain in the lower extremities such as osteoarthritis [23] and peripheral artery disease [4,12,24] cause people to restrict their activities of daily living and can lead to social isolation [25-27]. The GOALS intervention provided mastery experiences in which participants were encouraged to be active despite pain and discomfort, to observe the temporary nature of symptoms, and to soften into the localized and general muscular tension that accompanies these symptoms. They were encouraged to reflect on and take pride in their accomplishments. Self-regulatory skills were taught in a group-mediated setting that was nurturing, facilitated bonding between group members, and provided multiple opportunities for guidance.

While objective change in function is the primary medical objective of promoting physical activity for patients with PAD, what is often primary in the minds of older adults who choose to become more physically active are opportunities for social engagement, managing symptoms that accompany chronic disease, and preserving or enhancing their confidence with activities of daily living [28]. Indeed, such outcomes are critical to program adherence and to the promotion of well-being [29]. They may function as important mediators for long-term sustainability of gains resulting from lifestyle-related programs [30,31] and are vital to patient-centered care, a direction in medicine of rapidly growing importance.

## Conclusion

In summary, this is the first large scale trial to demonstrate that patients with PAD realize psychological and social benefits from a group-mediated home-based exercise program. The intervention group experienced greater improvement on self-efficacy, satisfaction with functioning, pain acceptance, and social functioning than an attention control group. Also, the effects were consistent across a number of important demographic, functional and disease-related variables.

## Abbreviations

PAD: Peripheral artery disease; GOALS: Group oriented arterial leg study; GMCB: Group-mediated cognitive behavioral intervention; ABI: Ankle brachial index; SPS: Social provision scale; RPE: Rating of perceived exertion; SD: Standard deviation; MI: Myocardial infarction; CHF: Congestive heart failure; BMI: Body mass index; LS: Least squared; Mdn: Median; BLV: Baseline visit; IC: Intermittent claudication; C: Control; I: Intervention.

## Competing interests

The authors declare that they have no competing interests.

## Authors’ contributions

JWR, MMM, and BS designed the study, developed the intervention and wrote the manuscript. HT, LT and LZ provided input on the study design, analyses and interpretation of the data. KD assisted with the development of the interventions, supervised collection of data, and was involved in writing the methods. All authors read and approved the final manuscript.
